# The Effect of Age Correction on Multivariate Classification in Alzheimer’s Disease, with a Focus on the Characteristics of Incorrectly and Correctly Classified Subjects

**DOI:** 10.1007/s10548-015-0455-1

**Published:** 2015-10-06

**Authors:** Farshad Falahati, Daniel Ferreira, Hilkka Soininen, Patrizia Mecocci, Bruno Vellas, Magda Tsolaki, Iwona Kłoszewska, Simon Lovestone, Maria Eriksdotter, Lars-Olof Wahlund, Andrew Simmons, Eric Westman, for the AddNeuroMed consortium and the Alzheimer’s Disease Neuroimaging Initiative

**Affiliations:** Department of Neurobiology, Care Sciences and Society, Karolinska Institutet, Novum, Plan 5, 141 57 Stockholm, Sweden; Department of Neurology, University of Eastern Finland and Kuopio University Hospital, Kuopio, Finland; Institute of Gerontology and Geriatrics, University of Perugia, Perugia, Italy; INSERM U 558, University of Toulouse, Toulouse, France; 3rd Department of Neurology, Medical School, Aristotle University of Thessaloniki, Thessaloniki, Greece; Medical University of Lodz, Lodz, Poland; Institute of Psychiatry, King’s College London, London, UK; NIHR Biomedical Research Centre for Mental Health, London, UK

**Keywords:** OPLS, Age correction, Alzheimer’s disease, Mild cognitive impairment, MRI, Early diagnosis, Diagnostic misclassification

## Abstract

The similarity of atrophy patterns in Alzheimer’s disease (AD) and in normal aging suggests age as a confounding factor in multivariate models that use structural magnetic resonance imaging (MRI) data. To study the effect and compare different age correction approaches on AD diagnosis and prediction of mild cognitive impairment (MCI) progression as well as investigate the characteristics of correctly and incorrectly classified subjects. Data from two multi-center cohorts were included in the study [AD = 297, MCI = 445, controls (CTL) = 340]. 34 cortical thickness and 21 subcortical volumetric measures were extracted from MRI. The age correction approaches involved: using age as a covariate to MRI-derived measures and linear detrending of age-related changes based on CTL measures. Orthogonal projections to latent structures was used to discriminate between AD and CTL subjects, and to predict MCI progression to AD, up to 36-months follow-up. Both age correction approaches improved models’ quality in terms of goodness of fit and goodness of prediction, as well as classification and prediction accuracies. The observed age associations in classification and prediction results were effectively eliminated after age correction. A detailed analysis of correctly and incorrectly classified subjects highlighted age associations in other factors: ApoE genotype, global cognitive impairment and gender. The two methods for age correction gave similar results and show that age can partially masks the influence of other aspects such as cognitive impairment, ApoE-e4 genotype and gender. Age-related brain atrophy may have a more important association with these factors than previously believed.

**Electronic supplementary material:**

The online version of this article (doi:10.1007/s10548-015-0455-1) contains supplementary material, which is available to authorized users.

## Introduction

Alzheimer’s disease (AD), the most common form of dementia, is a progressive neurodegenerative disorder that clinically characterizes by gradual loss of cognitive functions. Mild cognitive impairment (MCI), an intermediate condition between normal cognition and dementia, often represents a prodromal form of dementia. MCI patients have a significantly higher risk of converting to AD or other types of dementia. However not all MCI patients develop dementia even after several years. The new criteria for diagnosing “dementia due to AD” and “MCI due to AD” in addition to core clinical criteria, include the use of imaging and other biomarkers to improve the certainty of diagnoses (Albert et al. 2011; McKhann et al. 2011). However, the need of additional work to validate these biomarkers for routine clinical practice is also noted.

Structural magnetic resonance imaging (MRI) is an important marker in clinical practice for dementia diagnosis, particularly in memory clinic settings when younger and rare conditions are examined (Falahati et al. 2014a). MRI has been widely studied for early detection and diagnosis of AD in terms of atrophy of brain structures. In particular, atrophy of medial temporal structures such as hippocampus is demonstrated in AD patients (Scheltens et al. 1992). Due to the complexity of AD, measures of single structures from MRI are probably insufficient for accurate diagnosis. The combination of different structures has proven to be more useful when distinguishing AD from cognitively normal elderly subjects (CTL) (Westman et al. 2011b). With the help of sophisticated image analysis techniques, numerous volumetric and cortical thickness measures can be extracted from structural MRI data.

Machine learning and multivariate data analysis methods provide tools for processing and finding inherent patterns in such data with high complexity and dimensionality. Methods like orthogonal projection to latent structures (OPLS) (Bylesjö et al. 2006; Trygg and Wold 2002) are efficient, robust and validated tools for modeling complex biological data. OPLS was developed with the aim of reducing model complexity and improving model transparency. The improved interpretation property of the OPLS method postures it as a suitable analysis method. OPLS has successfully been applied in research for AD diagnosis and prediction of MCI progression (Aguilar et al. 2014; Spulber et al. 2013).

Confounding factors such as age negatively affect the performance of machine learning and multivariate models. Global and regional brain changes related to increasing age can potentially lead to misclassification of younger AD patients and older CTL subjects. Therefore, there is a need for developing methods to address this problem. Recently several methods for correcting the age associations are proposed (Dukart et al. 2011; Koikkalainen et al. 2012). The focus of these studies are statistical improvements while their effects on the characteristics of correctly and incorrectly classified subjects were disregarded. Studying the subjects’ characteristics is of high importance since it can distinctly reflect the way age correction improves the outcomes. Further, to compare these methods to simply use age as a covariate has not been properly investigated.

In this work two age correction approaches were investigated: simply using age as a variable in the OPLS model and a linear detrending approach that removes age-related effects from each variable based on measures in CTL subjects. The effect of age correction approaches on the classification of AD and CTL subjects, and prediction of progression from MCI to AD was explored. The characteristics of correctly/incorrectly classified and predicted subjects before and after age correction were studied in detail. We hypothesized that age correction would improve the performance of both classification and prediction. Additionally, studying the characteristics of subjects before and after age correction may reveal other clinically relevant aspects.

## Materials and Methods

### Study Setting

Data were obtained from two large multi-center cohorts, the Alzheimer’s disease Neuroimaging Initiative (ADNI) database (http://adni.loni.usc.edu) and AddNeuroMed. ADNI was launched in 2003 by the National Institute on Aging (NIA), the National Institute of Biomedical Imaging and Bioengineering (NIBIB), the Food and Drug Administration (FDA), private pharmaceutical companies and non-profit organizations (Mueller et al. 2005). The primary goal of ADNI is to test whether serial MRI, PET, other biological markers, and clinical and neuropsychological assessments can be combined to measure the progression of MCI and early AD. The Principal Investigator of this initiative is Michael W. Weiner, MD, VA Medical Center and University of California -San Francisco. ADNI subjects were recruited from over 50 sites across the U.S. and Canada. For up-to-date information, see www.adni-info.org. AddNeuroMed, a part of InnoMed (Innovative Medicines in Europe), is an integrated project aimed to develop and validate novel surrogate markers in AD (Lovestone et al. 2007). The neuroimaging part of AddNeuroMed uses MRI collected from six different sites across Europe (http://www.innomed-addneuromed.com/).

A total of 1082 subjects were included in the current study (AD = 297, MCI = 445 and CTL = 340). At 12-month follow-up, 85 MCI patients progressed to AD (MCI-p) and 360 remained stable (MCI-s). The demographics of the dataset are given in Table 1. The subjects in the ADNI study have also been followed up at 18, 24 and 36 months after baseline. MCI individuals who progressed to AD were considered as MCI-p and the rest as MCI-s.

**Table 1:** Demographic and clinical characteristics

	CTL	MCI-s	MCI-p^a^	AD
Count	340	360	85	297
Age, years	75.0 ± 5.7	75.0 ± 6.9	74.3 ± 6.5	75.7 ± 7.0
Gender, Female/Male	172/168	141/219	35/50	165/132
Education, years	14.3 ± 4.3	13.9 ± 4.7	13.8 ± 4.2	12.0 ± 4.9
MMSE score	29.1 ± 1.1	27.1 ± 1.7	26.5 ± 1.8	22.2 ± 3.7
CDR	0	0.5	0.5	0.92 ± 0.43
ApoE-e4, N/P	242/92	182/158	31/52	110/178
Cohort, ADNI/ANM	227/113	260/100	62/23	175/122

Continuous data is represented as mean ± SD, *CTL* control subjects, *MCI* mild cognitive impairment, *MCI-s* stable MCI, *MCI-p* progressive MCI, *AD* Alzheimer’s disease, *MMSE* mini mental state examination, *CDR* clinical dementia rating, *ApoE* apolipoprotein E, *N/P* negative/positive for at least one e4 allele, *ADNI/ANM* Alzheimer’s Disease Neuroimaging Initiative/AddNeuroMed

^a^MCI patients progressed to AD at month-12 follow-up

### Inclusion and Diagnostic Criteria

Participants’ recruitment and eligibility criteria were very similar in both cohorts (Petersen et al. 2010; Simmons et al. 2011). Briefly, AD diagnosis was based on NINCDS-ADRDA and DSM-IV criteria for probable AD, as well as a total clinical dementia rating (CDR) score of 0.5 or above. MCI diagnosis required a MMSE score between 24 and 30; memory complaints; normal activities of daily living; total CDR score of 0.5; and Geriatric Depression Scale (GDS) score of ≤5. The inclusion criteria for control participants were a MMSE score between 24 and 30; total CDR score of 0; and GDS score ≤5. No significant neurological or psychiatric illness, no significant unstable systemic illness or organ failure, and no history of alcohol or substance abuse or dependence were required for all three groups. MRI information was not used for diagnosis.

## Imaging

### MRI Data Acquisition

In both cohorts, 1.5T MRI data was collected from a variety of MR-systems with protocols optimized for each type of scanner. The MRI protocol included a high-resolution sagittal 3D T1-weighted MPRAGE volume (voxel size 1.1 × 1.1 × 1.2 mm^3^) acquired using a custom pulse sequence specifically designed for the ADNI study to ensure compatibility across scanners (Jack et al. 2008). MRI data acquisition in AddNeuroMed was designed to be compatible with the ADNI protocol (Simmons et al. 2011).

### Regional Subcortical Volume Segmentation and Cortical Thickness Parcellation

 The FreeSurfer pipeline (version 5.3.0) was applied to the MRI images to produce regional cortical thickness and subcortical volumetric measures. Full details and references of cortical reconstruction and subcortical volumetric segmentation procedure are included in the supplementary material 1. Data was processed through the hive database system (theHiveDB) (Muehlboeck et al. 2014). Visual quality control was performed on all output data. All steps involving brain extraction, automated Talairach transformation, tessellation, surfaces reconstruction, and subcortical segmentation were carefully checked. This segmentation approach has been used for multivariate classification of Alzheimer’s disease and healthy controls (Westman et al. 2010), neuropsychological-image analysis (Ferreira et al. 2014) and biomarker discovery (Maioli et al. 2015). In total, 55 MRI measures were used as input variables for OPLS classification, i.e. 34 regional cortical thickness measures and 21 regional subcortical volumes (measures from the left and right sides were averaged). Supplementary material 2 provides a list of measures and their mean and standard deviation in each diagnostic group. All subcortical volumetric and cortical thickness measures were used in their raw form (Westman et al. 2013).

## Data Analysis

### Multi and Univariate Data Analysis

Pre-processing was performed using mean-centering and unit variance scaling in order to transform the data into a suitable form for analysis (Eriksson et al. 2013). OPLS (Bylesjö et al. 2006; Trygg and Wold 2002), a supervised multivariate data analysis method, was used to classify AD patients and CTL individuals as well as to predict progression in the MCI patients. The OPLS method is an extension to the projection to latent structures (PLS) method (Wold et al. 1984). PLS has been developed for the purpose of modeling complex data based on the assumption that there are latent variables, which generate the observed data. PLS extracts these latent variables by maximizing the covariance between two sets of data, descriptor and response variables. In OPLS, the systematic variation in descriptor data is separated into two blocks, predictive variation correlated to response data and non-predictive variation orthogonal to response data. This separation improves the model transparency and reduces the model complexity. OPLS and PLS provide the same predictive accuracy, however, particularly for the two-class discriminant problem OPLS has an advantage over PLS that provides only one single predictive component (first component) and the other orthogonal components (if any) are not important in class separation. Accordingly, one single loading vector describes the class discriminating variables.

The performance of an OPLS model is quantified by two parameters, the goodness of fit (R^2^) and the goodness of prediction (Q^2^) (Eriksson et al. 2013). R^2^ is the fraction of variation of the training data that can be explained by the components of the model. R^2^ shows how well the model fits the training data. Q^2^ is the fraction of variation of the training data that can be predicted by the model. Q^2^ shows how reliable the model predicts new data. Q^2^ is used to find the optimal model complexity, which results in the most valid model with a balance between fit and predictive ability. Therefore, Q^2^ is more important than R^2^ and a model with higher Q^2^ is consider as a better model. Q^2^ is estimated by cross validation (CV). CV is a practical approach for evaluating learning algorithms that is based on building of a number of parallel models (Wold 1978). In this work, sevenfold CV was used to calculate Q^2^. In addition to Q^2^ and R^2^ as performance metrics, classification success rates were reported in terms of the accuracy, sensitivity and specificity.

For univariate comparisons of quantitative and qualitative variables, the independent samples *t* test and the χ^2^ test were used respectively.

### Age Correction Methods

Two age correction methods were implemented: (1) a simple approach that treats age as a covariate and includes age in the OPLS model as a separate variable along with MRI-derived variables and (2) a linear detrending algorithm based on age-related changes in the CTL group only. The detrending algorithm fits a generalized linear model (GLM) to each MRI-derived variable and age, in the CTL group only, and models the age-related changes as a linear drift. Then, the regression coefficient of the resulted GLM model (linear drift) is used to remove the age-related changes from all individuals (AD, MCI and CTL) and obtain corrected values. The linear model was chosen based on the Good et al. (2001) study where they found an age-related linear decrease in global grey matter volume in healthy individuals. The assumption for age correction method is that the age related changes in the CTL group are due to aging, while the age related changes in the MCI/AD group includes disease-related changes as well. Therefore, the algorithm calculates age-related effects based on the CTL group only, since removing age-related changes based on the AD or MCI group might also remove the disease-related changes. The detrending method was applied prior to further statistical analysis.

### Implementation

In the first step, three OPLS models were created for classification of AD and CTL subjects: (1) a model based on the raw measures (uncorrected model), (2) a model using age as a covariate (covariate model) and (3) a model based on age-detrended measures (detrended model). Subsequently, the resulted classification models were used to predict MCI patients as unseen data.

The output of the OPLS model is a cross-validated score vector where each score corresponds to one subject. A subject with a score close to one displays a pattern similar to AD and a subject with a score close to zero displays a pattern similar to CTL. A fixed cut-off equal to 0.5 was used to assign class membership to the predicted scores and afterwards to calculate accuracy, sensitivity and specificity (Spulber et al. 2013). Similarly, the prediction result for MCI patients is a score vector and by applying an appropriate cut-off (0.5), MCI patients can be predicted as CTL-like/AD-like. These steps were conducted for each age correction method.

All models were created hierarchically, i.e. volumetric and thickness measures were analyzed separately, and the output scores of these base models were used to create the final model. In the simple age correction method, age was included in the model along with base scores.

## Results

Table 2 summarizes the results from the analyses. The OPLS model based on the original MRI variables (uncorrected model) resulted in Q^2^ = 0.567 and R^2^ = 0.568. The model using original MRI data and age as a covariate (covariate model) resulted in Q^2^ = 0.580 and R^2^ = 0.586. The model based on age corrected MRI data (detrended model) resulted in Q^2^ = 0.582 and R^2^ = 0.583. The classification of AD and CTL subjects with uncorrected model resulted in an accuracy = 86.7 %, the covariate model resulted in an accuracy = 87.3 % and the detrended model resulted in an accuracy = 88.2 %. MCI prediction using the uncorrected model resulted in an accuracy = 62.7 %, the covariate model resulted in an accuracy = 62.9 % and finally the accuracy of the detrended model was 65.0 %.

**Table 2:** AD classification and MCI prediction results

	Model	Q^2^	R^2^	Accuracy %	Sensitivity %	Specificity %
AD versus CTL classification (CV)	Uncorrected	0.567	0.568	86.7	81.8	90.9
	Covariate	0.580	0.586	87.3	81.5	92.4
	Detrended	0.582	0.583	88.2	82.8	92.9
MCI prediction	Uncorrected	–	–	62.7	70.2	60.9
	Covariate	–	–	62.9	71.4	60.9
	Detrended	–	–	65.0	75.0	62.6

*AD* Alzheimer’s disease, *CTL* control subjects, *MCI* mild cognitive impairment, *CV* cross-validated, *Q* ^*2*^ goodness of prediction, *R* ^*2*^ goodness of fit

**Fig. 1:**
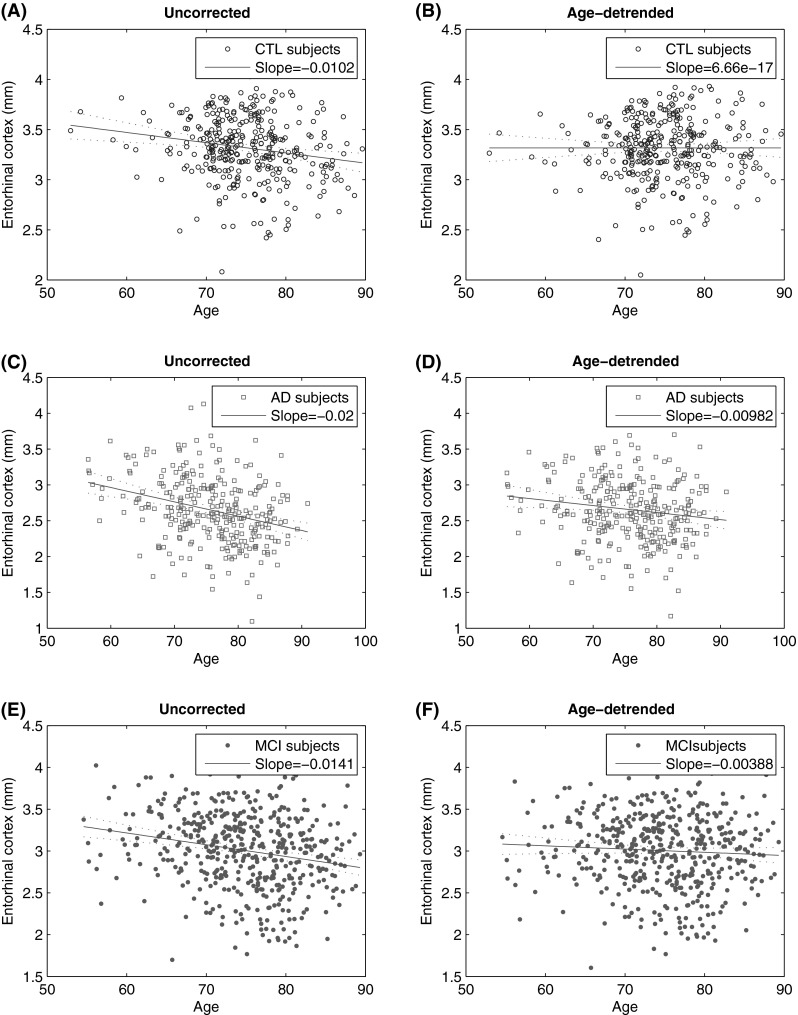
The *blue*, *green* and *cyan* markers represent the entorhinal cortex thickness of CTL, AD and MCI subjects before and after age correction with detrending method: **a** CTL subjects before age correction, **b** CTL subjects after age correction, **c** AD patients before age correction, **d** AD patients after age correction, **e** MCI patients before age correction and **f** MCI patients after age correction. Each marker corresponds to one subject. The *red lines* indicate the age-related drift fitted in the groups (Color figure online)

Medial temporal structures including amygdala, entorhinal cortex and hippocampus and the temporal gyrus regions (inferior, middle and superior) were the most important variables for the separation between the AD and CTL group in all three models. Figure 1 shows the thickness values of entorhinal cortex before and after applying age correction in the different groups. The Pearson correlation coefficients between all MRI measures and age, before and after age correction are given in supplementary material 3.

Table 3 shows the prediction results for the MCI subjects from the ADNI cohort at the different follow-up time points. At each time point, the detrended model resulted in the highest accuracy among the three models. In the uncorrected model, by increasing the follow-up duration from 12 months to 36 months, the prediction accuracy was improved from 60.9 to 66.8 %. In the detrended model, the prediction accuracy rose from 63.0 to 70.8 %.

**Table 3:** Prediction results for MCI subjects of ADNI cohort at different follow-up time points

Time point	Model	Accuracy %	Sensitivity %	Specificity %
Month-12 *MCI*-*p* = 62, *MCI*-*s* = 260	Uncorrected	60.9	72.1	58.2
	Covariate	60.2	70.5	57.9
	Detrended	63.0	75.4	60.2
Month-18 *MCI*-*p* = 90, *MCI*-*s* = 232	Uncorrected	64.9	72.2	62.1
	Covariate	66.1	74.4	62.9
	Detrended	67.7	75.6	64.7
Month-24 *MCI*-*p* = 126, *MCI*-*s* = 196	Uncorrected	65.5	66.7	64.8
	Covariate	67.4	69.0	66.3
	Detrended	68.9	69.8	68.4
Month-36 *MCI*-*p* = 150, *MCI*-*s* = 172	Uncorrected	66.8	65.3	68.0
	Covariate	69.3	68.0	70.3
	Detrended	70.8	68.7	72.7

*MCI* mild cognitive impairment, *MCI-s* stable MCI, *MCI-p* progressive MCI

Additional analyses were performed to further investigate the effect of the age correction methods on classification and prediction models. Table 4 shows the comparison between correct and incorrect classified subjects within each diagnostic group (i.e. within AD and CTL group) and the comparison of incorrect classified subjects between AD and CTL subjects. Without age correction, within both the CTL and the AD group, the mean age of correctly and incorrectly classified subjects was significantly different (*p* < 0.001 and *p* = 0.006, respectively). After accounting by age, both the covariate and detrended models showed no statistically significant age difference. The mean age of the incorrect classified CTL and AD subjects were 79.1 and 73.3 years, respectively (*p* < 0.001) in the uncorrected model. This difference was eliminated in both the covariate and detrended models. The MMSE score of AD subjects was significantly higher in incorrectly classified subjects in all three models. Moreover, the distribution of ApoE-e4 genotype was significantly different between incorrectly classified CTL and AD subjects.

**Table 4:** Subjects’ characteristics in AD versus CTL classification: comparison between correctly/incorrectly classified subjects

AD versus CTL Classification	CTL subjects			AD patients			Incorrect-classified AD versus CTL
	Correct-classified	Incorrect-classified	*p* value	Correct-classified	Incorrect-classified	*p* value	*p* value
*Uncorrected model*							
Count	309	31		243	54		
Age, years	74.5 ± 5.6	79.1 ± 5.2	**<0.001** ^**a**^	76.2 ± 6.7	73.3 ± 7.9	**0.006** ^**a**^	**<0.001** ^**a**^
Education, years	14.3 ± 4.3	14.1 ± 5.5	0.839^a^	11.9 ± 4.8	12.8 ± 5.1	0.197^a^	0.294^a^
MMSE score	29.1 ± 1.1	28.9 ± 1.2	0.326^a^	21.8 ± 3.7	24.2 ± 2.8	**<0.001** ^**a**^	**<0.001** ^**a**^
Gender, Male/Female	152/157	16/15	0.797^b^	107/136	25/29	0.762^b^	0.637^b^
ApoE-e4, N/P	217/87	26/5	0.138^b^	86/149	24/29	0.240^b^	**0.001** ^b^
Cohort, ADNI/ANM	207/102	20/11	0.780^b^	141/102	34/20	0.505^b^	0.886^b^
*Covariate model*							
Count	314	26		242	55		
Age, years	74.8 ± 5.7	77.0 ± 4.3	0.058^a^	75.7 ± 6.8	75.4 ± 7.7	0.776^a^	0.237^a^
Education, years	14.2 ± 4.2	14.0 ± 5.6	0.781^a^	11.9 ± 4.7	12.5 ± 5.3	0.412^a^	0.269^a^
MMSE score	29.1 ± 1.1	28.9 ± 1.1	0.326^a^	21.7 ± 3.7	24.4 ± 2.7	**<0.001** ^**a**^	**<0.001** ^**a**^
Gender, Male/Female	153/161	15/11	0.380^b^	103/139	29/26	0.171^b^	0.675^b^
ApoE-e4, N/P	223/84	20/5	0.385^b^	83/151	27/27	**0.048** ^b^	**0.012** ^b^
Cohort, ADNI/ANM	210/104	17/9	0.876^b^	143/99	32/23	0.902^b^	0.536^b^
*Detrended model*							
Count	316	24		246	51		
Age, years	75.0 ± 5.8	75.3 ± 4.6	0.756^a^	75.5 ± 7.0	76.3 ± 7.1	0.493^a^	0.553^a^
Education, years	14.4 ± 4.3	12.8 ± 5.6	0.205^a^	12.0 ± 4.8	12.4 ± 5.0	0.530^a^	0.756^a^
MMSE score	29.2 ± 1.1	28.8 ± 1.4	0.168^a^	21.8 ± 3.7	24.4 ± 2.7	**<0.001** ^**a**^	**<0.001** ^**a**^
Gender, Male/Female	155/161	13/11	0.629^b^	105/141	27/24	0.180^b^	0.921^b^
ApoE-e4, N/P	225/87	18/5	0.524^b^	86/151	24/27	0.156^b^	**0.012** ^b^
Cohort, ADNI/ANM	213/103	14/10	0.363^b^	144/102	31/20	0.767^b^	0.840^b^

*p* value in bold indicates statistically significant difference

Continuous data is represented as mean ± standard deviation, *AD* Alzheimer’s disease, *CTL* control subjects, *ApoE* apolipoprotein E, *N/P* negative/positive for at least one e4 allele, *MMSE* mini mental state examination, *ADNI/ANM* Alzheimer’s disease Neuroimaging Initiative/AddNeuroMed

^a^Independent-samples *t* test

^b^Pearson χ^2^

Table 5 shows the comparison between correctly and incorrectly predicted MCI patients within each group (i.e. within MCI-s and MCI-p group) as well as the comparison of incorrectly predicted subjects between MCI-s and MCI-p group. In MCI-s subjects, the mean age of correctly and incorrectly predicted subjects was significantly different without considering age (*p* < 0.001) but not after accounting for age. Using uncorrected data resulted in 3.9 years difference between the mean-age of misclassified MCI-p and MCI-s (*p* = 0.003), where incorrectly predicted MCI-s patients were older than incorrectly classified MCI-p subjects (77.0 and 73.1 years respectively). This difference was eliminated after age correction. Moreover, incorrectly predicted MCI-s patients showed significantly lower MMSE score and lower frequency of male in all three models. In addition, age correction led to a significant difference in ApoE-e4 distribution in MCI-s subjects, showing lower frequency of ApoE-e4 allele in incorrectly predicted subjects.

**Table 5:** Subjects’ characteristics in MCI prediction: comparison between correctly/incorrectly classified subjects

MCI prediction		MCI-s subjects			MCI-p subjects			Incorrect-predicted MCI-s versus MCI-p
		Correct-predicted	Incorrect-predicted	*p* value	Correct-predicted	Incorrect-predicted	*p* value	*p* value
Uncorrected model	Count	219	141		59	26		
	Age, years	73.6 ± 7.1	77.0 ± 6.1	**<0.001** ^**a**^	75.0 ± 6.9	73.1 ± 5.3	0.220^a^	**0.003** ^**a**^
	Education, years	13.8 ± 4.6	14.1 ± 4.7	0.582^a^	13.8 ± 4.4	13.7 ± 3.8	0.820^a^	0.651^a^
	MMSE score	27.4 ± 1.7	26.8 ± 1.6	**0.001** ^**a**^	26.5 ± 1.9	26.6 ± 1.6	0.802^a^	0.682^a^
	Gender, Male/Female	144/75	75/66	**0.017** ^**b**^	35/24	15/11	0.888^**b**^	0.672^**b**^
	ApoE-e4, N/P	118/87	65/71	0.077^**b**^	22/36	8/16	0.694^**b**^	0.190^**b**^
	Cohort, ADNI/ANM	151/68	109/32	0.084^**b**^	44/15	18/8	0.609^**b**^	0.375^**b**^
Covariate model	Count	219	141		60	25		
	Age, years	74.7 ± 7.1	75.3 ± 6.6	0.419^a^	74.3 ± 7.0	74.6 ± 5.0	0.852^a^	0.635^a^
	Education, years	13.8 ± 4.6	14.2 ± 4.8	0.394^a^	13.9 ± 4.4	13.7 ± 3.9	0.853^a^	0.614^a^
	MMSE score	27.4 ± 1.7	26.8 ± 1.7	**0.001** ^**a**^	26.6 ± 1.9	26.4 ± 1.6	0.740^a^	0.383^a^
	Gender, Male/Female	149/70	70/71	**<0.001** ^**b**^	37/23	13/12	0.409^**b**^	0.828^**b**^
	ApoE-e4, N/P	122/83	61/75	**0.008** ^**b**^	20/39	10/13	0.418^**b**^	0.902^**b**^
	Cohort, ADNI/ANM	150/69	110/31	0.050^**b**^	43/17	19/6	0.682^**b**^	0.824^**b**^
Detrended model	Count	225	135		63	22		
	Age, years	75.2 ± 7.1	74.4 ± 6.6	0.273^a^	74.1 ± 6.9	75.4 ± 4.9	0.408^a^	0.492^a^
	Education, years	13.8 ± 4.5	14.2 ± 4.8	0.478^a^	14.0 ± 4.1	13.2 ± 4.5	0.418^T^	0.380^a^
	MMSE score	27.4 ± 1.7	26.7 ± 1.7	**0.001** ^**a**^	26.5 ± 1.9	26.6 ± 1.6	0.775^a^	0.803^a^
	Gender, Male/Female	154/71	65/70	**<0.001** ^**b**^	39/24	11/11	0.329^**b**^	0.872^**b**^
	ApoE-e4, N/P	127/83	56/75	**0.001** ^**b**^	40/22	12/8	0.715^**b**^	0.817^**b**^
	Cohort, ADNI/ANM	156/69	104/31	0.114^**b**^	46/17	16/6	0.979^**b**^	0.659^**b**^

*p* value in bold indicates statistically significant difference

Continuous data is represented as mean ± SD, *MCI* mild cognitive impairment, *MCI-p* progressive MCI, *MCI-s* stable MCI, *ApoE* apolipoprotein E, *N/P* negative/positive for at least one e4 allele, *MMSE* mini mental state examination, *ADNI/ANM* Alzheimer’s disease Neuroimaging Initiative/AddNeuroMed

^a^Independent-samples *t* test

^b^Pearson χ^2^

## Discussion

In recent years, there has been an increased interest in using advanced machine learning and multivariate data analysis methods and structural MRI data for early diagnosis of AD. Notably, the discriminative capacity of MRI-derived features and several classifiers for classifying AD patients and CTL individuals and for predicting progression from MCI to AD has been investigated (Liu et al. 2013; Wee et al. 2013; Wolz et al. 2011). The OPLS method in this work resulted in high classification accuracy and good prediction outcomes. OPLS has previously been used for classification purposes in the same two multi-center cohorts considered here (Westman et al. 2011a). Despite the input data (dataset subjects) and image processing (FreeSurfer software version) are non-identical in the two studies, the accuracy levels were analogous. It has previously been shown that different advanced classifiers applied to the same data provide similar levels of accuracy (Aguilar et al. 2013). At present, limitations are probably related to input data (quality of data or cohort studied), clinical diagnosis or the confounding effect of some demographic variables such as age, rather than the method used for classification (Falahati et al. 2014b).

Age as a confounding factor can negatively affect the classification and prediction performance. Indeed, the similarity of atrophy patterns in AD patients and in cognitively normal subjects can lead to misclassification of young AD patients and old CTL subjects. Global and regional changes of brain volumes in normal aging have been reported in cross-sectional and longitudinal brain imaging studies (Giorgio et al. 2010; Good et al. 2001; Scahill et al. 2003; Walhovd et al. 2005). Additionally, several studies have reported that brain atrophy accelerates with disease progression in AD and other types of dementia (Fox et al. 1996; Jack et al. 2004; Sabuncu et al. 2011; Tisserand et al. 2004). Particularly, global brain atrophy and reduced volume in the temporal lobe especially in hippocampus and entorhinal cortex have been reported.

Using age as a covariate in statistical models is a common way to deal with this problem. Recently, new approaches such as a data correction method based on linear regression models (Dukart et al. 2011; Koikkalainen et al. 2012), and confounder correcting support vector machine algorithm (Li et al. 2011) have been proposed to more effectively control for the effect of age as a confounding factor. In this study, two approaches i.e. age as a covariate and deterending age-related changes were investigated. Both studied approaches here have pros and cons. In heterogeneous populations containing AD, MCI and CTL subjects with different patterns and rates of atrophy simply using age as a covariate may not be an optimal approach. However, the OPLS method seems to be able to handle age as a covariate. Detrending age-related changes is challenging since modeling the exact association between age and discriminative features and subsequently remove such associations can be difficult and time consuming. One of the main ideas behind the detrending method was to remove age-related changes while preserving the disease-related changes for each variable separately. The hypothesis was that by detrending the AD and the MCI group based on CTL group, the age-related changes would be omitted and the disease-related changes would be kept. Therefore the control group should be representative of population and equally distributed on the age range. Hence the detrending method may be more effective in a larger dataset. In some variables (e.g. hippocampus) the slopes for MCI and AD were slightly tilted in the opposite direction after age correction, indicating that the algorithm may overcorrect in older AD and MCI cases. A reduction in rates of atrophy in older AD and MCI compared to CTL could be a possible explanation for the observed pattern. A recent study has shown that a pronounced reduction in rates of atrophy can be observed in AD and MCI individuals with increasing age, while for cognitively normal individuals, increasing age leads to increased rates of atrophy (Holland et al. 2012).

The results indicate that when age is included, the quality of models in terms of the goodness of fit and more importantly the goodness of prediction was improved which led to higher classification and predication accuracies. Although accurate discrimination between AD patients and CTL subjects is of great interest, prediction of progression form MCI to AD is more valuable since it can provide an opportunity for early detection of individuals under risk of developing dementia. Generally, MCI prediction accuracy is not as good as classification of AD and CTL subjects. Reviewing the literature, classification accuracies tend to range between 80 and 90 %, mostly accompanied by lower prediction accuracies for MCI progression (Falahati et al. 2014b). The MCI group is clinically quite heterogeneous. Some MCI subjects progress to AD or even other neurological disorders, some remain stable over time, with a smaller number reverting to a cognitively normal status (Mitchell and Shiri-Feshki 2009). In addition, one-year is a relatively short follow-up time. When the subjects were followed for a longer period, the accuracy increased. Including age in the models also improved prediction accuracies. Although, the covariate and detrended models performed similarly in terms of model quality values, the detrended model induced the highest accuracies in all settings.

A detailed analysis of correctly classified and incorrectly classified subjects provided valuable information on the age correction performance. As expected, younger AD patients and older CTL subjects were more likely to be misclassified. Similarly, younger MCI-p and older MCI-s patients were prone to misclassification. These findings are in line with previous studies (Dukart et al. 2011).

Incorrectly classified AD subjects had significantly higher MMSE score compared to correctly classified AD subjects. This can be explained by the previous finding that in AD patients, decreased MMSE score correlates with gray matter reduction (Frisoni et al. 2002) and correlates with aging (Pradier et al. 2014). In MCI-s subjects, the mean MMSE score was slightly higher in correctly classified subjects.

Interestingly, incorrectly classified MCI-s subjects were more frequently ApoE-e4 positive compared to correctly classified MCI-s (Table 5). This is in line with previous studies that reported more regional atrophy in AD patients with presence of genetic risk, especially in the medial temporal cortex (Cherbuin et al. 2007; Ferreira et al. 2015; van der Flier et al. 2011). The incorrectly classified MCI-s subjects may thus have a high risk of developing AD in the future.

The frequency of misclassification was higher in female MCI-s subjects compared to males, indicating that more female subjects had AD-like structural patterns. This is in line with recent findings that the female sex is associated with an increased risk of disease progression (Tifratene et al. 2015). Although this difference exists even before age correction, after correcting for age, more female subjects were prone to be misclassified which could emphases the role of age. This can support the age- by sex- related differences in progression rates that proposed in several studies (Mielke et al. 2014; Roberts et al. 2014).

The characteristics of correctly and incorrectly classified/predicted subjects were similar for both correction approaches. In fact, the association between structural brain changes, age, sex, ApoE genotype, cognitive status and other factors are more complicated than pairwise relations. Although the relationship between these factors are explored from several perspectives such as age by sex relations (Fratiglioni et al. 1997), sex by ApoE genotype relation (Altmann et al. 2014), etc., the connection between them is poorly understood. Considering age in multivariate models, regardless of approach, can potentially enhance the outcomes.

## Conclusion

Both age correction approaches (age as a covariate and detrending) could effectively eliminate the age differences in classification and prediction results. Moreover, including age in the models highlighted the role of the other disease-related factors such as cognitive impairment and ApoE-e4 genotype. These results demonstrate that age is partially masking other relevant factors such as ApoE genotype, global cognitive impairment and sex. This is an important finding, suggesting that mechanisms underlying the confounding effect of these factors should be further investigated. At the time being, clinicians are already quite aware about the effect of age when interpreting imaging data for diagnostic purposes. Therefore, the other factors should also be carefully considered when adjusting diagnostic interpretations of imaging data in clinical settings. The exact relationship between normal ageing and AD is far from being fully understood at present and warrants further investigations. Non-linear correction methods and other alternatives for handling confounding factors should be further investigated. Applying correction methods to other confounding factors such as education and sex would be of interest and could potentially improve prediction accuracy of MCI progression further.

## Electronic supplementary material

Supplementary material 1 (PDF 64 kb)

Supplementary material 2 (PDF 85 kb)

Supplementary material 3 (PDF 97 kb)
