# Supplementary material for: The Effect of Age Correction on Multivariate Classification in Alzheimer’s Disease, with a Focus on the Characteristics of Incorrectly and Correctly Classified Subjects
Source: Brain Topogr. 2015 Oct 6;29:296–307. doi: 10.1007/s10548-015-0455-1 (PMC4754326; doi:10.1007/s10548-015-0455-1)
Supplement: Supplementary file 1 — Supplementary material 1 (PDF 64 kb) [file 10548_2015_455_MOESM1_ESM.pdf]

Supplementary material 1

Cortical reconstruction and subcortical volumetric segmentation includes removal of non-brain tissue using a hybrid watershed/surface deformation procedure (Segonne et al. 2004), automated Talairach transformation, segmentation of the subcortical white matter and deep grey matter volumetric structures (including hippocampus, amygdala, caudate, putamen, ventricles) (Fischl et al. 2002; Fischl et al. 2004; Segonne et al. 2004) intensity normalization (Sled et al. 1998), tessellation of the grey matter white matter boundary, automated topology correction (Fischl et al. 2001; Segonne et al. 2007), and surface deformation following intensity gradients to optimally place the grey/white and grey/cerebrospinal fluid borders at the location where the greatest shift in intensity defines the transition to the other tissue class (Dale and Sereno 1993; Fischl and Dale 2000; Fischl et al. 1999). Once the cortical models are complete, registration to a spherical atlas takes place which utilizes individual cortical folding patterns to match cortical geometry across subjects (Fischl et al. 1999). This is followed by parcellation of the cerebral cortex into units based on gyral and sulcal structure (Desikan et al. 2006; Fischl et al. 2004). The pipeline generated 68 cortical thickness measures (34 from each hemisphere) and 50 regional subcortical volumes. Volumes of white matter hypointensities, optic chiasm, right and left vessel, and left and right choroid plexus were excluded from further analysis. White matter hypointensities were excluded since most subjects were characterized by zero values. Cortical thickness and subcortical volumetric measures from the right and left side were averaged, since this makes the data interpretation easier and the prediction accuracy does not significantly change if measures are averaged.

References

Dale AM, Sereno MI (1993) Improved Localizadon of Cortical Activity by Combining EEG and MEG with MRI Cortical Surface Reconstruction: A Linear Approach Journal of cognitive neuroscience 5:162-176 doi:10.1162/jocn.1993.5.2.162

Desikan RS et al. (2006) An automated labeling system for subdividing the human cerebral cortex on MRI scans into gyral based regions of interest Neuroimage 31:968-980 doi:10.1016/j.neuroimage.2006.01.021

Fischl B, Dale AM (2000) Measuring the thickness of the human cerebral cortex from magnetic resonance images Proceedings of the National Academy of Sciences of the United States of America 97:11050-11055 doi:10.1073/pnas.200033797

Fischl B, Liu A, Dale AM (2001) Automated manifold surgery: constructing geometrically accurate and topologically correct models of the human cerebral cortex IEEE Trans Med Imaging 20:70-80 doi:10.1109/42.906426

Fischl B et al. (2002) Whole brain segmentation: automated labeling of neuroanatomical structures in the human brain Neuron 33:341-355 doi:10.1016/S0896-6273(02)00569-X

Fischl B, Salat DH, van der Kouwe AJ, Makris N, Segonne F, Quinn BT, Dale AM (2004) Sequence-independent segmentation of magnetic resonance images Neuroimage 23 Suppl 1:S69-84 doi:10.1016/j.neuroimage.2004.07.016

Fischl B, Sereno MI, Tootell RB, Dale AM (1999) High-resolution intersubject averaging and a coordinate system for the cortical surface Hum Brain Mapp 8:272-284 doi:10.1002/(SICI)1097-0193(1999)8:4<272::AID-HBM10>3.0.CO;2-4

Segonne F, Dale AM, Busa E, Glessner M, Salat D, Hahn HK, Fischl B (2004) A hybrid approach to the skull stripping problem in MRI Neuroimage 22:1060-1075 doi:10.1016/j.neuroimage.2004.03.032

Segonne F, Pacheco J, Fischl B (2007) Geometrically accurate topology-correction of cortical surfaces using nonseparating loops IEEE Trans Med Imaging 26:518-529 doi:10.1109/TMI.2006.887364

Sled JG, Zijdenbos AP, Evans AC (1998) A nonparametric method for automatic correction of intensity nonuniformity in MRI data IEEE Trans Med Imaging 17:87-97 doi:10.1109/42.668698
