# Supplementary material for: The Effect of Age Correction on Multivariate Classification in Alzheimer’s Disease, with a Focus on the Characteristics of Incorrectly and Correctly Classified Subjects
Source: Brain Topogr. 2015 Oct 6;29:296–307. doi: 10.1007/s10548-015-0455-1 (PMC4754326; doi:10.1007/s10548-015-0455-1)
Supplement: Supplementary file 2 — Supplementary material 2 (PDF 85 kb) [file 10548_2015_455_MOESM2_ESM.pdf]

Supplementary material 2

Mean and standard deviation of MRI measures within different diagnosis groups.

		CTL (n=340)	MCI-s (n=360)	MCI-p (n=85)	AD (n=297)
Volumetric Measures (mm^3)	**3rd Ventricle**	1812 ± 682	1909 ± 700	1981 ± 667	2150 ± 735
	**4th Ventricle**	1844 ± 687	1789 ± 658	1780 ± 619	1807 ± 648
	**Accumbens area**	399 ± 86	381 ± 90	349 ± 72	329 ± 76
	**Amygdala**	1289 ± 188	1198 ± 231	1045 ± 202	1017 ± 222
	**Brain Stem**	20443 ± 2248	20447 ± 2504	20239 ± 2288	19727 ± 2385
	**Caudate**	3441 ± 529	3496 ± 601	3373 ± 502	3400 ± 658
	**Cerebellum cortex**	45935 ± 4689	46137 ± 5478	45947 ± 4891	44825 ± 5317
	**Cerebellum white matter**	12590 ± 1734	12777 ± 2028	12381 ± 1850	12227 ± 1869
	**Corpus callosum anterior**	718 ± 132	710 ± 148	702 ± 124	667 ± 133
	**Corpus callosum central**	340 ± 62	331 ± 63	326 ± 61	309 ± 59
	**Corpus callosum midanterior**	337 ± 68	325 ± 72	321 ± 73	303 ± 68
	**Corpus callosum midposterior**	316 ± 75	302 ± 69	294 ± 68	277 ± 57
	**Corpus callosum Posterior**	863 ± 145	853 ± 152	860 ± 153	797 ± 139
	**Hippocampus**	3544 ± 470	3191 ± 562	2819 ± 512	2660 ± 537
	**Inferior lateral ventricle**	814 ± 551	1109 ± 720	1406 ± 739	1710 ± 1036
	**Lateral ventricle**	17149 ± 9481	20448 ± 10865	22559 ± 10006	23990 ± 12163
	**Pallidum**	1385 ± 174	1402 ± 185	1377 ± 175	1328 ± 186
	**Putamen**	4502 ± 587	4458 ± 624	4348 ± 527	4225 ± 658
	**Thalamus proper**	6264 ± 662	6252 ± 754	6186 ± 677	6004 ± 751
	**Sulcal CSF**	1228 ± 345	1352 ± 385	1453 ± 450	1472 ± 477
	**Ventral diencephalon**	3329 ± 376	3325 ± 404	3238 ± 347	3158 ± 384
Thickness measures (mm)	**Banks of superior temporal sulcus**	2.25 ± 0.16	2.18 ± 0.17	2.12 ± 0.19	2.06 ± 0.2
	**Caudal anterior cingulate gyrus**	2.54 ± 0.23	2.51 ± 0.24	2.53 ± 0.28	2.53 ± 0.27
	**Caudal middle frontal gyrus**	2.29 ± 0.16	2.22 ± 0.17	2.2 ± 0.17	2.15 ± 0.2
	**Cuneus cortex**	1.62 ± 0.1	1.62 ± 0.12	1.6 ± 0.11	1.6 ± 0.13
	**Entorhinal cortex**	3.32 ± 0.33	3.06 ± 0.44	2.79 ± 0.48	2.65 ± 0.47
	**Frontal pole**	2.6 ± 0.24	2.54 ± 0.25	2.5 ± 0.25	2.46 ± 0.28
	**Fusiform gyrus**	2.48 ± 0.15	2.4 ± 0.16	2.33 ± 0.18	2.25 ± 0.21
	**Inferior parietal cortex**	2.16 ± 0.14	2.09 ± 0.16	2.04 ± 0.18	1.98 ± 0.19
	**Inferior temporal gyrus**	2.63 ± 0.15	2.55 ± 0.17	2.45 ± 0.19	2.39 ± 0.23
	**Insular cortex**	2.81 ± 0.16	2.75 ± 0.18	2.71 ± 0.17	2.65 ± 0.23
	**Isthmus cingulate cortex**	2.15 ± 0.17	2.1 ± 0.18	2.06 ± 0.17	2 ± 0.2
	**Lateral occipital cortex**	1.95 ± 0.13	1.91 ± 0.13	1.88 ± 0.14	1.85 ± 0.17
	**Lateral orbito frontal cortex**	2.44 ± 0.15	2.4 ± 0.16	2.38 ± 0.16	2.32 ± 0.19
	**Lingual gyrus**	1.75 ± 0.1	1.73 ± 0.11	1.73 ± 0.11	1.68 ± 0.12
	**Medial orbito frontal cortex**	2.27 ± 0.15	2.23 ± 0.17	2.18 ± 0.17	2.14 ± 0.19
	**Middle temporal gyrus**	2.64 ± 0.14	2.54 ± 0.17	2.46 ± 0.19	2.39 ± 0.22
	**Paracentral sulcus**	2.07 ± 0.18	2.02 ± 0.18	2.02 ± 0.19	2.01 ± 0.22
	**Parahippocampal gyrus**	2.51 ± 0.28	2.34 ± 0.32	2.27 ± 0.33	2.17 ± 0.33
	**Parsopercularis gyrus**	2.32 ± 0.15	2.28 ± 0.16	2.27 ± 0.15	2.21 ± 0.19

**Parsorbitalis gyrus**	2.52 ± 0.19	2.46 ± 0.2	2.45 ± 0.19	2.39 ± 0.22
**Parstriangularis gyrus**	2.19 ± 0.15	2.14 ± 0.15	2.13 ± 0.17	2.1 ± 0.17
**Pericalcarine cortex**	1.38 ± 0.08	1.38 ± 0.11	1.36 ± 0.1	1.36 ± 0.11
**Postcentral gyrus**	1.8 ± 0.13	1.77 ± 0.13	1.76 ± 0.15	1.72 ± 0.14
**Posterior cingulate gyrus**	2.29 ± 0.15	2.23 ± 0.14	2.21 ± 0.16	2.18 ± 0.18
**Precentral gyrus**	2.19 ± 0.18	2.13 ± 0.19	2.15 ± 0.21	2.08 ± 0.21
**Precuneus cortex**	2.05 ± 0.14	2 ± 0.15	1.96 ± 0.16	1.91 ± 0.18
**Rostral anterior cingulate gyrus**	2.72 ± 0.2	2.68 ± 0.22	2.67 ± 0.23	2.65 ± 0.26
**Rostral middle frontal gyrus**	2.15 ± 0.14	2.1 ± 0.15	2.07 ± 0.15	2.02 ± 0.16
**Superior frontal gyrus**	2.47 ± 0.16	2.4 ± 0.16	2.38 ± 0.17	2.34 ± 0.19
**Superior parietal gyrus**	1.91 ± 0.14	1.87 ± 0.16	1.84 ± 0.15	1.8 ± 0.19
**Superior temporal gyrus**	2.5 ± 0.16	2.42 ± 0.17	2.35 ± 0.19	2.29 ± 0.21
**Supramarginal gyrus**	2.24 ± 0.15	2.19 ± 0.16	2.16 ± 0.18	2.09 ± 0.19
**Temporal pole**	3.56 ± 0.29	3.39 ± 0.35	3.22 ± 0.37	3.17 ± 0.41
**Transverse temporal gyrus**	2.08 ± 0.23	2.04 ± 0.23	2.03 ± 0.26	1.96 ± 0.29

Data is represented as mean ± standard deviation, *CTL* control subjects, *MCI* mild cognitive impairment, *MCI-s* stable MCI, *MCI-p* progressive MCI, *AD* Alzheimer's disease, *CSF* cerebrospinal fluid.
