# Supplementary material for: The Effect of Age Correction on Multivariate Classification in Alzheimer’s Disease, with a Focus on the Characteristics of Incorrectly and Correctly Classified Subjects
Source: Brain Topogr. 2015 Oct 6;29:296–307. doi: 10.1007/s10548-015-0455-1 (PMC4754326; doi:10.1007/s10548-015-0455-1)
Supplement: Supplementary file 3 — Supplementary material 3 (PDF 97 kb) [file 10548_2015_455_MOESM3_ESM.pdf]

Supplementary material 3

Pearson correlation coefficients between MRI measures and age, before and after age correction.

	CTL		MCI		AD	
	Original	Age-corrected	Original	Age-corrected	Original	Age-corrected
**3rd Ventricle**	0.325**	0	0.298**	-0.090*	0.276**	-0.096
**4th Ventricle**	0.150**	0	0.068	-0.124**	0.136*	-0.059
**Accumbens area**	-0.282**	0	-0.341**	-0.002	-0.211**	0.181**
**Amygdala**	-0.272**	0	-0.141**	0.128**	-0.144*	0.137*
**Brain Stem**	-0.119*	0	-0.212**	-0.083	-0.170**	-0.035
**Caudate**	0.166**	0	0.098*	-0.086	0.228**	0.067
**Cerebellum cortex**	-0.146**	0	-0.295**	-0.144**	-0.262**	-0.109
**Cerebellum white matter**	-0.227**	0	-0.341**	-0.109*	-0.316**	-0.062
**Corpus callosum anterior**	-0.300**	0	-0.329**	0.014	-0.301**	0.065
**Corpus callosum central**	-0.231**	0	-0.326**	-0.046	-0.310**	-0.016
**Corpus callosum midanterior**	-0.304**	0	-0.352**	-0.001	-0.265**	0.106
**Corpus callosum midposterior**	-0.262**	0	-0.285**	0.067	-0.214**	0.212**
**Corpus callosum Posterior**	-0.136*	0	-0.227**	-0.069	-0.179**	-0.005
**Hippocampus**	-0.426**	0	-0.369**	0.066	-0.326**	0.135*
**Inferior lateral ventricle**	0.371**	0	0.357**	0.025	0.317**	0.08
**Lateral ventricle**	0.330**	0	0.300**	-0.048	0.265**	-0.051
**Pallidum**	-0.064	0	-0.155**	-0.082	-0.204**	-0.132*
**Putamen**	-0.034	0	-0.141**	-0.102*	-0.059	-0.022
**Thalamus proper**	-0.217**	0	-0.307**	-0.076	-0.229**	0.004
**Sulcal CSF**	0.225**	0	0.216**	-0.015	0.185**	-0.014
**Ventral diencephalon**	-0.163**	0	-0.236**	-0.049	-0.309**	-0.118*
**Banks of superior temporal sulcus**	-0.176**	0	-0.113*	0.072	0.041	0.206**
**Caudal anterior cingulate gyrus**	0.117*	0	0.053	-0.08	0.074	-0.046
**Caudal middle frontal gyrus**	-0.180**	0	-0.218**	-0.002	-0.069	0.115*
**Cuneus cortex**	-0.125*	0	-0.108*	0.026	-0.099	0.02
**Entorhinal cortex**	-0.179**	0	-0.216**	-0.061	-0.298**	-0.152**
**Frontal pole**	-0.171**	0	-0.178**	0.025	-0.185**	-0.004
**Fusiform gyrus**	-0.255**	0	-0.216**	0.072	-0.185**	0.039
**Inferior parietal cortex**	-0.262**	0	-0.147**	0.134**	0.046	0.279**
**Inferior temporal gyrus**	-0.202**	0	-0.205**	0	-0.087	0.073
**Insular cortex**	-0.155**	0	-0.139**	0.035	-0.149**	-0.014
**Isthmus cingulate cortex**	-0.170**	0	-0.139**	0.069	-0.043	0.139*
**Lateral occipital cortex**	-0.284**	0	-0.244**	0.092*	-0.107	0.161**
**Lateral orbito frontal cortex**	-0.224**	0	-0.174**	0.08	-0.197**	0.016
**Lingual gyrus**	-0.225**	0	-0.220**	0.041	-0.153**	0.085
**Medial orbito frontal cortex**	-0.108*	0	-0.134**	-0.02	-0.219**	-0.121*
**Middle temporal gyrus**	-0.215**	0	-0.172**	0.036	-0.113	0.054
**Paracentral sulcus**	-0.142**	0	-0.200**	-0.023	-0.136*	0.007
**Parahippocampal gyrus**	-0.175**	0	-0.145**	0.043	-0.215**	-0.033

**Parsopercularis gyrus**	-0.133*	0	-0.193**	-0.038	-0.135*	0
**Parsorbitalis gyrus**	-0.168**	0	-0.183**	0.005	-0.194**	-0.024
**Parstriangularis gyrus**	-0.133*	0	-0.254**	-0.103*	-0.230**	-0.09
**Pericalcarine cortex**	-0.125*	0	-0.108*	0.006	-0.047	0.072
**Postcentral gyrus**	-0.124*	0	-0.251**	-0.103*	-0.195**	-0.057
**Posterior cingulate gyrus**	-0.099	0	-0.046	0.076	-0.026	0.073
**Precentral gyrus**	-0.218**	0	-0.308**	-0.062	-0.231**	-0.008
**Precuneus cortex**	-0.180**	0	-0.155**	0.049	-0.001	0.164**
**Rostral anterior cingulate gyrus**	0.042	0	-0.05	-0.096*	-0.108	-0.148*
**Rostral middle frontal gyrus**	-0.185**	0	-0.257**	-0.043	-0.143*	0.058
**Superior frontal gyrus**	-0.288**	0	-0.287**	0.066	-0.207**	0.095
**Superior parietal gyrus**	-0.142**	0	-0.137**	0.02	-0.024	0.108
**Superior temporal gyrus**	-0.365**	0	-0.324**	0.075	-0.296**	0.028
**Supramarginal gyrus**	-0.222**	0	-0.219**	0.035	-0.105	0.113
**Temporal pole**	-0.234**	0	-0.191**	0.045	-0.255**	-0.054
**Transverse temporal gyrus**	-0.131*	0	-0.250**	-0.100*	-0.287**	-0.166**

CTL control subjects, *MCI* mild cognitive impairment, *AD* Alzheimer's disease, *CSF=cerebrospinal fluid.*

* Correlation is significant at the 0.05 level (2-tailed)

** Correlation is significant at the 0.01 level (2-tailed).
